# Pre-operative hemoglobin level and use of sedative-hypnotics are independent risk factors for post-operative delirium following total knee arthroplasty

**DOI:** 10.1186/s12891-020-03206-4

**Published:** 2020-05-02

**Authors:** Eiji Kijima, Tomohiro Kayama, Mitsuru Saito, Daisaburo Kurosaka, Ryo Ikeda, Hiroteru Hayashi, Daisuke Kubota, Takashi Hyakutake, Keishi Marumo

**Affiliations:** Department of Orthopaedic Surgery, The Jikei University School of Medicine, 3-25-8 Nishi-Shimbashi, Minato-ku, Tokyo, 105-8461 Japan

**Keywords:** Total knee arthroplasty, Delirium, Sedative-hypnotics, Post-operative complications, Anemia, Orthopaedic surgery, Elderly

## Abstract

**Background:**

Delirium is a well-known complication following surgery, especially with the increasing age of patients undergoing surgery. The increasing demands resulting from a prolonged healthy life expectancy has resulted in more arthroplasties despite their age and existing comorbidities. The purpose of this study is to explore the various risk factors that may contribute to delirium in unilateral and bilateral total knee arthroplasties in the elderly population.

**Methods:**

170 patients who underwent unilateral or bilateral total knee arthroplasties were analyzed retrospectively for delirium. Age, sex, comorbidities, use of sedative-hypnotics, peri-operative blood loss, pre- and post-operative laboratory blood test results were investigated and analyzed.

**Results:**

The incidence of post-operative delirium was 6.5% (11 out of 170 patients) with a mean age of 79.5 (± 6.9) years, compared to 73.0 (± 9.0) years in the non-delirium group. Higher age, use of sedative-hypnotics, low pre-operative Hb and Ht, low post-operative Hb, Ht and BUN were observed in the delirium group. Multivariate logistic regression analysis identified that the use of sedative-hypnotics and pre-operative Hb level were independent risk factors for post-operative delirium after TKA. The odds ratios for the use of sedative-hypnotics and pre-operative Hb level were 4.6 and 0.53, respectively. Receiver operating characteristic curve analysis showed that pre-operative Hb of less than 11.1 g/dL was a predictor for the development of delirium, with a sensitivity of 54.6% and a specificity of 91.6%.

**Conclusion:**

Patients with a pre-operative Hb level of < 11.1 g/dL or those using sedative-hypnotics are associated with post-operative delirium. Peri-operative management and preventative measures are therefore needed to reduce the risks of post-operative delirium in such patients.

## Background

Delirium is an acute brain dysfunction characterized by disorganized thinking and attention deficit, in addition to mild-to-moderate disturbance of consciousness [1]. The development of delirium is thought to be related to decreased oxygen supply and electrolyte imbalance. The onset is typically acute and the symptoms show marked diurnal fluctuation. The accompanying symptoms include characteristic hallucination, illusion, disorientation, and abnormal behavior [2]. Post-operative delirium is a serious condition associated with adverse clinical and economic outcomes, including higher rates of major complications, decreased cognitive function, poor functional recovery, increased length of stay, increased mortality, and higher costs [3–6]. In the acute clinical setting, it is thought that 70% of older patients have some degree of cognitive impairment [7]. More specifically delirium is estimated to be present in up to 30% of hospitalized patients [8]. The incidence of post-operative delirium in the field of Orthopaedics is reported to be 0.59–20%, with old age, anemia and poor nutrition the risk factors for post-operative delirium [3, 9–18]. In the Asian population who underwent TKA, history of dementia, history of cerebrovascular disease, low BMI, chronic opioid use, large decrease in post-operative Hb and albumin, and post-operative high BUN have been associated with post-operative delirium [9, 14, 18]. A systematic review by Bin Abd Razak et al. have identified age, history of psychiatric illness, decreased functional status and decreased verbal memory as independent predictors of delirium in total joint arthroplasties. Additionally, the use of isofluorane for general anaesthesia and benzodiazepines have been implicated while cognitive assessment has been found to be a useful predictor for post-operative delirium [19].

The problems faced by patients with post-operative delirium after total knee arthroplasty (TKA) include increased risks of falls and fractures due to continued rest, delayed rehabilitation, and prolonged hospital stay. The identification of risk factors for post-operative delirium is therefore important for the prevention and peri-operative management of patients undergoing elective surgery.

In recent years, the number of elderly patients undergoing TKA has increased, both in terms of age and numbers. However, few studies have investigated the incidence of post-operative delirium after TKA, and its risk factors have not been fully identified. Currently, there is no hospital protocol to identify patients at risk of delirium or methods to prevent its occurrence. The objective of this study was to investigate the incidence of post-operative delirium, associated comorbidities, and peri-operative risk factors for patients after TKA. The result would assist in identifying patients at risk, and aims to prevent the development of delirium post-operatively.

## Methods

A total of 170 consecutive patients who underwent unilateral or bilateral TKA in our hospital between June 2013 and April 2015 were included in this study. There were 32 men and 138 women with a mean age of 74 years (range, 37 to 95 years). This gender ratio of those who underwent primary TKA was in keeping with the ratio from the national registry [20]. 92 patients underwent unilateral TKA, whereas 78 underwent bilateral TKA. The diagnoses at surgery were as follows: knee osteoarthritis in 154 patients, rheumatoid arthritis in 8 patients, spontaneous osteonecrosis of the knee in 4 patients, and psoriatic arthritis in 1 patient.

Delirium was screened and diagnosed using the Confusion Assessment Method (CAM), and the Diagnostic and Statistical Manual 5 diagnostic criteria (DSM 5) [21–23]. (Table 1).

**Table 1:** Diagnostic criteria for delirium. Confusion Assessment Method (CAM, adapted from Inouye et al. [23]) and Diagnostic and Statistical Manual of Mental Disorders 5 (DSM-5, adapted from Diagnostic and Statistical Manual of Mental Disorders, fifth edition [24].) were used to diagnose delirium

**Confusion Assessment Method (CAM)**	
- Clinical features 1 and 2 are required in addition to either 3 or 4.	
Feature 1: Acute onset and fluctuating course of mental status	
Feature 2: Inattention	
Feature 3: Disorganized thinking	
Feature 4: Altered level of consciousness	
**Diagnostic and Statistical Manual of Mental Disorders (DSM-5)**	
- All criteria (A-E) must be met.	
A. Disturbance in attention and awareness	
B. Acute onset with fluctuation in severity	
C. An additional disturbance in cognition	
D. The disturbances are not better explained by pre-existing cognitive disorders	
E. There is evidence of a direct physiological consequence or multiple etiologies	

Assessment was based on written medical records, entered by physicians and nurses. Two qualified medical doctors checked independently that the symptoms met both CAM and DSM-5 criteria for delirium, and were only diagnosed as delirium when the two assessors were in consensus. The time of onset, duration, and symptoms of post-operative delirium were recorded. To identify the risk factors for post-operative delirium, the patients were divided into two groups: those who developed delirium (delirium group) and those who did not (non-delirium group).

The investigated factors included age, sex, type of surgery (unilateral or bilateral TKA), history of hypertension, diabetes mellitus, ischemic heart disease, use of sedative-hypnotics, amount of peri-operative blood loss, pre- and post-operative day 1 and day 3 laboratory blood test results (white blood cells, hemoglobin [Hb], hematocrit [Ht], total protein, albumin, blood urea nitrogen [BUN], creatinine, sodium, potassium, chloride and calcium).

Standard hospital protocol for TKA surgery was performed for all patients, and routine post-operative rehabilitation was commenced for all patients. After surgery, the patients were mobilized in a wheelchair on post-operative day 1 and commenced standing and gait exercise (depending on pain) from post-operative day 2.

Patients received the following pain control regimens: Sciatic nerve block (0.375% ropivacaine 20 ml) or epidural block (0.25% ropivacaine 4 ml/hr) was used unless they declined or if the block risk was deemed too high. Celecoxib 200 mg was administered orally twice a day from post-operative day 1 until post-operative day 14. Patients with chronic kidney disease received acetaminophen 600 mg three times a day instead of celecoxib. Stronger painkillers such as tramadol were administered if post-operative pain was not controlled adequately with the above methods.

The statistical analyses performed were as follows. The nominal values were compared using the chi-squared test and the continuous variables, using the analysis of variance (ANOVA) test. The statistical significance was set at *P* < 0.05. Of the factors with significance, age, pre-operative Hb, use of sedative-hypnotics, and post-operative day 3 BUN were selected for multivariate analysis, based on significance and previous reports. Logistic multiple regression analysis was performed to identify independent risk factors for delirium.

All protocols in this study are under the approval of the ethics committee for clinical research at The Jikei University School of Medicine (Approval number: 31–222(9721)).

## Results

The incidence of post-operative delirium was 6.5% (11 out of 170 patients). The time of onset ranged from the day of surgery to post-operative day 3 (mean, 0.8 days). 6 patients (55%) developed delirium on the day of surgery, 3 patients (27%) on post-operative day 1, and 1 patient each (9%) on post-operative day 2 and day 3. The duration of delirium ranged from 1 day to 5 days (mean duration 1.8 days). The most prominent symptoms of delirium were as follows: “disorientation” in 7 patients (64%), “unintelligible speech and behavior” in 4 patients (36%), “hallucinations, such as auditory/visual hallucination” in 2 patients (18%), “difficulty resting at night (for example, sitting or standing at night)” in 2 patients (18%), and “yelling” in 1 patient (9%). One patient required medical treatment by a psychiatrist (Table 2).

**Table 2:** Symptoms of delirium. Main symptoms, time of onset and duration of symptoms are listed. Disorientation was observed in 7 out of 11 patients, and hallucination and unintelligible speech were also observed in several patients. The onset of symptoms was mostly observed either on the day of surgery or 1 day post-operatively, although 1 case each of delayed onset was observed on days 2 and 3. Duration of delirium was usually short but 2 patients had symptoms persisting for more than 3 days

Patient No.	Main Symptoms	Time of onset	Duration
1	Disorientation	Post-operative day 3	2days
2	Disorientation	Day of surgery	1day
3	Disorientation, unintelligible speech and behavior	Post-operative day 1	1day
4	Unintelligible speech and behavior	Day of surgery	1day
5	Disorientation	Day of surgery	1day
6	Visual hallucination, talkativeness, standing at night	Day of surgery	3days
7	Auditory hallucination, sitting up at night	Day of surgery	5days
8	Disorientation, unintelligible speech and behavior	Post-operative day 1	1day
9	Unintelligible speech and behavior, raising voice at night	Day of surgery	1day
10	Disorientation	Post-operative day 1	1day
11	Disorientation requiring psychiatric intervention	Post-operative day 2	2days

The mean age was 79.5 (± 6.9) years in the delirium group and 73.0 (± 9.0) years in the non-delirium group; the delirium group comprised of significantly older individuals than the non-delirium group (*P* = 0.021). The sex-wise incidence of post-operative delirium was 6.3% (2 out of 32 patients) among men and 6.5% (9 out of 138 patients) among women, with no significant difference between the sexes (*P* = 0.955). The incidence according to the type of surgery was 4.3% (4 out of 92 patients) for unilateral TKA and 9.0% (7 out of 78 patients) for bilateral TKA, with no significant difference between the methods (*P* = 0.221).

The number of regular users of sedative-hypnotics was greater in the delirium group with 5 out of 11 patients (45%), compared to 27 out of 159 patients (17.0%) in the non-delirium group (*P* = 0.036). All patients were prescribed benzodiazepine receptor agonists, except for 1 patient who was prescribed ramelton, a melatonin receptor, and did not develop delirium. There was 1 patient with pre-operative dementia, but post-operative delirium was not observed in this patient. 1 patient had a previous history of delirium, and this patient developed delirium post-operatively. There were also isolated cases of post-operative complications such as a cerebrovascular event and a seizure, but again, they were not observed in the delirium group. Significant differences were also observed in patients with low pre-operative levels of Hb (*P* < 0.001) and Ht (*P* < 0.001). Low post-operative day 1 levels of Hb (*P* = 0.028), Ht (*P* = 0.034), and post-operative day 3 BUN levels (*P* = 0.008) were also observed in the delirium group. There were no significant differences in sex, type of surgery (unilateral or bilateral), amount of blood loss, history of hypertension, diabetes mellitus, ischemic heart disease, or other blood test results between the delirium and non-delirium groups (Table 3).

**Table 3:** Assessment of risk factors for delirium. Patient characteristics were stratified by post-operative delirium status. Significant difference was observed for age, use of sedative-hypnotics, pre-operative Hb, post-operative day 1 Hb, pre-operative Ht, post-operative day 1 Ht, and post-operative day 3 BUN

	Delirium (***n*** = 11)	No delirium (***n*** = 159)	***p***-Value
**Age (years)**	79.5 ± 6.9	73.0 ± 9.0	0.021*
Sex (male / female)	2/9	30/129	0.955
Type of surgery (unilateral / bilateral TKA)	4/7	88/71	0.221
History of hypertension (n) (%)	9 (81.8%)	86 (54.1%)	0.061
History of diabetes (n) (%)	2 (18.2%)	25 (15.7%)	0.832
History of ischemic heart disease (n) (%)	0 (0%)	5 (3.1%)	0.410
**Use of sedative-hypnotics (n) (%)**	5 (45.5%)	27 (17.0%)	0.036*
Estimated blood loss (ml)	543 ±169	741 ±594	0.273
**Pre-operative levels of Hb (g/dl)**	11.6 ±1.7	13.2 ±1.4	<0.001*
**Post-operative day 1 Hb (g/dl)**	9.9 ±1.6	11.0 ±1.4	0.028*
Post-operative day 3 Hb (g/dl)	8.9 ±1.5	10.4 ±7.7	0.519
**Pre-operative levels of Ht (%)**	34.8 ±4.7	39.7 ±3.9	<0.001*
**Post-operative day 1 Ht (%)**	30.4 ±4.7	33.2 ±4.2	0.034*
Post-operative day 3 Ht (%)	26.9 ±4.6	29.5 ±4.8	0.077
Post-operative day 1 TP (g/dl)	5.89 ±0.3	5.68 ±0.5	0.184
Post-operative day 3 TP (g/dl)	5.49 ±0.34	5.54 ±0.57	0.775
Post-operative day 1 Alb (g/dl)	3.28 ±0.17	3.21 ±0.30	0.443
Post-operative day 3 Alb (g/dl)	2.76 ±0.29	2.80 ±0.36	0.766
Post-operative day 1 BUN (mg/dl)	14.9 ±6.4	14.2 ±7.2	0.763
**Post-operative day 3 BUN (mg/dl)**	20.5 ±8.3	14.2 ±7.3	0.008*
Post-operative day 1 Cr (mg/dl)	0.80 ±0.29	0.80 ±1.33	0.987
Post-operative day 3 Cr (mg/dl)	0.89 ±0.28	0.80 ±1.40	0.835
Post-operative day 1 Na (mmol/l)	138.7 ±3.4	139.0 ±2.4	0.740
Post-operative day 3 Na (mmol/l)	138.6 ±2.8	140.0 ±3.0	0.147
Post-operative day 1 K (mmol/l)	4.15 ±0.59	4.27 ±3.40	0.908
Post-operative day 3 K (mmol/l)	3.88 ±0.39	3.90 ±0.41	0.868
Post-operative day 1 Cl (mmol/l)	105.6 ±3.2	105.5 ±2.6	0.966
Post-operative day 3 Cl (mmol/l)	105.3 ±3.0	105.9 ±3.2	0.623
Post-operative day 1 Ca (mg/dl)	8.65 ±0.64	8.36 ±0.36	0.307
Post-operative day 3 Ca (mg/dl)	8.90 ±0.41	8.39 ±0.15	0.281

**Fig. 1:**
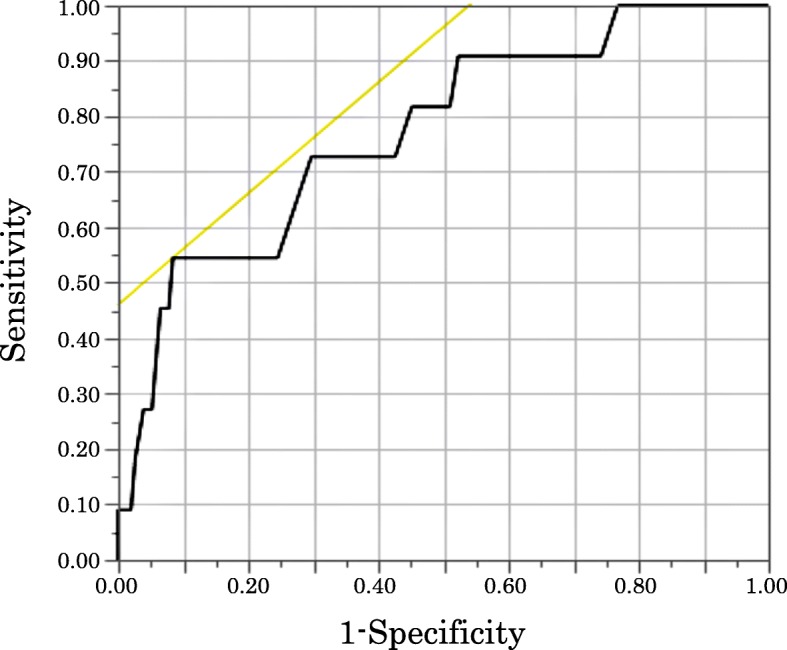
Receiver operating characteristic (ROC) curve of post-operative day 1 Hb for delirium. ROC curve analysis of pre-operative Hb level as a predictor for the development of delirium showed that the pre-operative Hb cut-off level was 11.1 g/dL, with a sensitivity of 54.6% and a specificity of 91.6% (AUC value 0.77)

Multivariate logistic regression analysis was performed for the variables that were significantly associated with post-operative delirium on univariate analysis (Table 4). The use of sedative-hypnotics and pre-operative Hb level were identified as independent risk factors for post-operative delirium after TKA. The adjusted odds ratios for the use of sedative-hypnotics and pre-operative Hb level were 4.6 and 0.53, respectively. Receiver operating characteristic (ROC) curve analysis showed that pre-operative Hb of less than 11.1 g/dL was a predictor for the development of delirium, with a sensitivity of 54.6% and a specificity of 91.6% (AUC value 0.77) (Fig. 1).

**Table 4:** Multivariate logistic regression analysis of risk factors of delirium. Use of sedative-hypnotics and pre-operative Hb level were independent risk factors for developing post-operative delirium. The adjusted odds ratios for the use of sedative-hypnotics and pre-operative Hb level were 4.6 and 0.53, respectively

	Odds ratio	95% CI	*P*-Value
Age (years)	1.08	0.99-1.20	0.08
**Use of sedative-hypnotics**	**4.61**	**1.11-20.2**	0.035^*^
**Pre-operative levels of Hb (g/dl)**	**0.53**	**0.31-0.87**	0.011^*^
Post-operative day 3 BUN (mg/dl)	1.05	0.98-1.13	0.171

## Discussion

The presence of delirium increases the risk of developing dementia later in life [25]. Although generally a temporal condition presenting with psychiatric symptoms, delirium can lead to decreased quality of life and reduced ability to perform routine activities; therefore, its prevention and treatment are important, especially in elective surgery.

Delirium is generally induced by organic brain damage, systemic diseases, and pharmacological factors. The causes of delirium are often difficult to identify, and multiple factors are believed to be involved in its pathogenesis. The most well known risk factors are history of delirium (odds ratio, 4.1), advanced age (≥70 years) (odds ratio, 3.2), and pre-existing cognitive dysfunction before surgery (odds ratio, 2.2) [26]. Other reported risk factors for delirium include low Hb or Ht, malnutrition, and dehydration, by causing low blood pressure and metabolic imbalance leading to hypoxic brain injury [11, 27]. It was also reported that the risk of delirium increased 1.15-fold per year increase in age [9]. As the number of elderly patients undergoing TKA has increased in recent years, attention to incidence of post-operative delirium is warranted in clinical practice, as this affects time until ambulation and duration of hospital stay [14]. This study consisted of patients with a higher average age compared to previous studies, and also included a larger number of bilateral TKA [9, 14]. In this study, the incidence of post-operative delirium after TKA was 6.5%, similar to previous reports, and no difference was observed between unilateral and bilateral TKA patients, despite bilateral TKA consisting of longer surgery time and increased peri-operative blood loss.

The pathogenesis of delirium is complex and multifactorial, and the utilization of multivariate analysis is a useful approach for identifying significant risk factors. Multivariate analysis of risk factors for post-operative delirium after TKA identified the use of sedative-hypnotics and a pre-operative Hb level of < 11.1 g/dL as independent risk factors. The use of sedative-hypnotics increased the risk of delirium by more than 4 times. Age, Hb/Ht level on post-operative day 1, and BUN level on post-operative day 3 showed significant differences in the univariate analysis but not in the multivariate analysis. Given that the Hb/Ht and BUN levels are associated with dehydration and anemia, further analysis is required to assess their true association with delirium.

In recent years, there have been reports that benzodiazepine receptor agonists (benzodiazepines and non-benzodiazepines) may cause cognitive decline, mental dependence, delirium, and muscle relaxation, with increased risks of falls and proximal femoral fractures [15, 28, 29]. New insomnia drugs, such as ramelteon and suvorexant, act selectively on the pineal and hypothalamic receptors (melatonin receptor and orexin receptor), respectively, potentially reducing the risk of delirium. Furthermore, Hatta et al. reported in a randomized clinical trial, that ramelteon and suvorexant even showed suppressive effects against delirium [30, 31]. Therefore if possible, switching from benzodiazepine receptor agonists to these drugs prior to surgery may have a preventative effect for pre-operative elderly patients against delirium.

There has been a growing interest in the role of anemia in cognition among older adults. Recent systematic review and meta-analysis showed significant positive association between anemia and global cognitive decline, reduced executive function, and the incidence of dementia [19, 32]. A recent report suggested anemia as an independent risk factor for delirium in hospitalized older patients along with dementia in a small cohort of 190 patients [33]. However, in a more recent multicenter study, no evidence of association was found between anemia and cognitive impairment [34].

Tranexamic acid has been shown to decrease intra-operative blood loss and the need for overall blood transfusion in knee and hip arthroplasties, and its efficacy and safety has been evaluated in a meta-analysis [35–37]. However, pre-operative anemia remains a risk-factor for blood transfusion despite tranexamic acid administration, and attempts to minimize post-operative Hb decrease have not been sufficient to compensate for low pre-operative Hb [38]. Furthermore, pre-operative blood transfusion remains controversial, with reports of multiple complications associated with pre-operative blood transfusion in hip arthroplasty [39].

Oral iron supplement has not been proven to be beneficial, due to its low absorption rate and gastrointestinal side-effects [40]. Intravenous iron therapy on the other hand is a more effective method with fewer side effects. It reduced the rate of transfusion and length of hospital stay, but there is no conclusive evidence at present to suggest that correcting pre-operative anemia results in reduced risk of delirium post-operatively [41].

Although no definitive preventative measures for delirium exist, its multi-factorial nature suggests that for elderly patients with either low Hb or sedative-hypnotics, particular consideration is necessary to reduce other potential risk factors such as peri-operative bleeding and dehydration. Current hospital practice involves minimizing blood loss intra-operatively by administering tranexamic acid and avoiding dehydration by administration of intravenous fluids peri-operatively. We encourage early rehabilitation and gait exercise to prevent prolonged bed stay to reduce post-operative complications. However, no specific measures had been taken to identify and reduce the risks of delirium. As a result, previous pre-operative assessment included rigorous medical assessment, but lacked cognitive assessment and did not take into account the use of pre-operative benzodiazepine receptors. Taking into account the findings from this study, we are developing a new hospital protocol, to be particularly cautious in patients with Hb < 11.1 g/dl, and explaining the risks of delirium to patients and their family. Furthermore, we are screening for patients who are taking benzodiazepine receptor agonists, and where possible, switching them to alternative sedative-hypnotics such as melatonin and orexin receptors prior to surgery. In addition, administration of benzodiazepine receptor agonists are avoided post-operatively.

There were several limitations in this study. First, the study design was retrospective, and therefore the details of delirium could only be assessed based on the existing medical records. More importantly, pre-operative cognitive function was not assessed, which is an indicator of post-operative delirium. A detailed analysis is required to assess the specific risk of TKA in cognitive decline. Another limitation was that the number of patients in the delirium group was much smaller than that in the non-delirium group. There are also reports of subsyndromal delirium, which are not easily identified, but may be significant in terms of risks of falls and development of future dementia [42, 43]. Again, these may be identified by pre-operative cognitive function screening in a future study.

A prospective study in future with the accrual of more cases and routine delirium assessment may further our understanding of the risk factors for delirium.

## Conclusion

Post-operative delirium is a significant surgical complication affecting both short-term in-hospital care and long-term outcome. It is the responsibility of the surgeon to minimize the risks of post-operative delirium using appropriate prevention and management, particularly in elective surgery. Results of this study showed that patients with a pre-operative Hb level of < 11.1 g/dL and those using sedative-hypnotics are associated with post-operative delirium. Peri-operative management and preventative measures are therefore needed to reduce the risks of post-operative delirium in such patients.

## Data Availability

The datasets used and/or analyzed during the current study are available from the corresponding author on reasonable request.

## Abbreviations

TKA: Total knee arthroplasty
CAM: Confusion Assessment Method
DSM 5: Diagnostic and Statistical Manual 5 diagnostic criteria
Hb: Hemoglobin
Ht: Hematocrit
BUN: Blood urea nitrogen
